# Phospholipase A2 of Peroxiredoxin 6 Plays a Critical Role in Cerebral Ischemia/Reperfusion Inflammatory Injury

**DOI:** 10.3389/fncel.2017.00099

**Published:** 2017-04-05

**Authors:** Yu Shanshan, Jiang Beibei, Tan Li, Gao Minna, Lei Shipeng, Peng Li, Zhao Yong

**Affiliations:** ^1^Department of Pathology, Chongqing Medical UniversityChongqing, China; ^2^Molecular Medical Laboratory, Chongqing Medical UniversityChongqing, China; ^3^Institute of Neuroscience, Chongqing Medical UniversityChongqing, China; ^4^Key Laboratory of Neurobiology, Chongqing Medical UniversityChongqing, China; ^5^Department of Respiratory Medicine, Jiangjin Center HospitalChongqing, China

**Keywords:** Prdx6-iPLA2 activity, cerebral ischemia/reperfusion, OGD/R, microglia/neuron co-culture system, MJ33

## Abstract

Microglia-mediated inflammation is an important step in the progression of cerebral ischemia/reperfusion injury and the associated production of receptors of immunomoudulation, including Toll-like receptors (TLRs). Peroxiredoxin 6 (Prdx6) has been demonstrated as the endogenous antioxidant protein for its peroxidase properties. However, the role of the independent phospholipase A2 (iPLA2) activity of Prdx6 in stroke has not been well studied. In this study, we evaluated whether blocking the calcium-iPLA2 activity of Prdx6 using siRNA and inhibitors (1-hexadecyl-3-(trifluoroethgl)-sn-glycerol-2 phosphomethanol, MJ33) would have a critical effect on inflammatory brain damage. We conducted oxygen-glucose deprivation (OGD)/recovery (R) *in vitro* and middle cerebral artery occlusion (MCAO) *in vivo* in a microglia/neuron co-culture system and in rats. *In vitro*, we found that Prdx6-iPLA2 activity was associated with the secretion of neurotoxic inflammatory mediators interleukin1β (IL-1β), interleukin-17 (IL-17) and interleukin-23 (IL-23) and elevated expression of Toll-like receptor 2/4 (TLR2/4), leading to the formation of nuclear factor-kappa B (NF-κB), inducible nitric oxide synthase (iNOS) and cyclooxygenase-2 (COX-2) in microglial cells. *In vivo*, combined treatment with Prdx6-iPLA2 activity inhibitor MJ33 showed a greater diminution in neurologic deficits, cerebral infarction, brain water content and inflammatory molecules than Prdx6-siRNA treatment alone. Our findings provide new insight into Prdx6-iPLA2 function in the brain. Inhibition of Prdx6-iPLA2 activity by gene therapy and/or pharmacology may constitute a promising new therapeutic approach to the treatment of stroke.

## Introduction

Despite the complex pathogenesis of ischemic stroke, emerging evidence suggests that inflammation is an important step in the primary and secondary progression of cerebral ischemia/reperfusion injury (Shichita et al., 2012a,b; Kuang et al., 2014; Yu et al., 2015). Microglia in the brain plays a prominent role in initiating, sustaining and resolving post-ischemic inflammation (Chen et al., 2011; Benakis et al., 2004). It has previously been recognized that inhibiting the activation of immunomoudulation receptors, including Toll-like receptors (TLRs) caused by microglial phagocytosis could prevent inflammatory neuronal death (Neher et al., 2011; Kuang et al., 2014). Among the TLRs, TLR2 and TLR4 are mainly expressed in the microglia of the brain and have been found to be more important than others in the immune response of the microglia (Wang et al., 2013; Lv et al., 2016). Therefore, the mechanisms that underlie microglia-neuron crosstalk are under extensive investigation for the development of innovative neuroprotective therapies for ischemic stroke.

Peroxiredoxin 6 (Prdx6) is the uniquely 1-Cys member of the peroxiredoxin family with both GSH peroxidase and calcium-independent phospholipase A2 (iPLA2) activities (Manevich and Fisher, 2005). Although the GSH peroxidase activity of Prdx6 has been widely studied in cell and animal models for its antioxidant function, the function of the iPLA2 activity of Prdx6 remains unclear. With loss- and gain-of-function mutations of Prdx6, Kim et al. (2011) found that the restoration of Prdx6 without iPLA2-mutant resulted in dramatic recovery of tumor necrosis factor-induced apoptosis. Blocking Prdx6-iPLA2 activity by its specific inhibitor 1-hexadecyl-3-(trifluoroethgl)-sn-glycerol-2 phosphomethanol (MJ33) could significantly protect the lung against damage from hyperoxia (Benipal et al., 2015). Recently, Prdx6-iPLA2 activity has been reported as giving off potent proinflammatory signals (Garcia-Bonilla and Iadecola, 2012; Shichita et al., 2012a,b). In human bronchial epithelial cells (BEAS2B), the production of interleukin-1β (IL-1β) was dependent on the iPLA2 activity of Prdx6 (Kim et al., 2011). Lee et al. (2013) reported that Prdx6-iPLA2 activity was associated with lung inflammation through activation of NADPH oxidase (NOX2). In primary cultured astrocytes, iPLA2 activity of Prdx6 induced astrocytic activation followed by increased proinflammatory cytokines (tumor necrosis factor-α (TNF-α) and IL-1β (Yun et al., 2015). All of these results strongly suggest that the iPLA2 activity of Prdx6 may be involved in the proinflammatory stimuli. However, until now, no study has described the iPLA2 activity of the Prdx6 function in microglia-mediated neuroinflammation and cerebral ischemia/reperfusion.

Based on previous research, we hypothesized that the iPLA2 activity of Prdx6 has a critical role in cerebral ischemia/reperfusion inflammatory injury. To explore the molecular mechanism that underlines the critical effect of Prdx6-iPLA2 activity, we conducted oxygen-glucose deprivation/recovery (OGD/R) *in vitro* and middle cerebral artery occlusion (MCAO) *in vivo*, in a microgila/neuron co-culture system and in rats, respectively. siRNA and inhibitors (MJ33) were used to inhibit Prdx6-iPLA2 enzymatic activity.

## Materials and Methods

### Rat Primary Microglia/Neuron Co-Culture System

Microglia and neurons were derived from 1-day-old Sprague-Dawley rats. All experimental protocols were approved by the Institutional Animal Care and Use Committee of Chongqing Medical University, China. All efforts were made to minimize suffering. Microglia-enriched cells were obtained by the method of Suzumura et al. (1987). After maintenance of mixed glial cultures for 9–12 days, microglial cells were separated by shaking flasks for 1 h using a rotary shaker at 200 rpm. The isolated cells were grown in DMEM/F12 supplemented with 10% FBS and 1% penicillin/streptomycin. When the microglia’s growth density reached 55%–60%, cortex neuron-enriched cultures were obtained according to our previously published methods (Chen et al., 2016; Li et al., 2016). The purity of the primary neuron cultures was over 90%, as determined by neuron-specific marker NeuN.

The rat primary microglia/neuron co-culture system was performed as described previously with some changes (Correa et al., 2013; Bi et al., 2014). Briefly, a Transwell co-culture system utilized the non-contact Transwell inserts. Then the primary microglia and neurons were co-cultured for a period of 24 h, sharing the same culture medium containing 2% B27 and 1% penicillin/streptomycin through a 0.4-μm transmembrane that prevented cell migration but allowed small molecule exchange.

### Oxygen Glucose Deprivation and Regeneration (OGD/R) and Groups

To simulate ischemic conditions, cultures were exposed to OGD/R as we previously described (Chen et al., 2016; Li et al., 2016). The medium was replaced by Neurobasal media without glucose and the cells were placed in an incubator with 1% O_2_, 94% N_2_ and 5% CO_2_ concentration. After a 6 h OGD, reoxygenation was carried out by transferring the cells to the normal cell culture incubator for 24 h. The co-culture systems were divided into five groups: (1) control group (Control): microglia without any treatment; (2) OGD/R group (OGD/R); (3) scramble group (Scramble): microglia treated with a scramble of siRNA and subjected to OGD/R; (4) Prdx6-iPLA2 activity siRNA group (Prdx6-iPLA2 siRNA): microglia transfected with Prdx6-iPLA2 siRNA and subjected to OGD/R; and (5) MJ33 group (MJ33): microglia given MJ33 treatment (50 μmol/L) and subjected to OGD/R.

### Prdx6-iPLA2 siRNA

Lentivirus was supplied by Neuron Biotech (Shanghai, China). In order to create siRNA in the iPLA2 activity of Prdx6, we knocked down Prdx6 with lentivirus (NCBI accession no. NM_053576.2 → NP-446028.1; shRNA, 5′-ACAGCCCGTGTGGTATTCAT-3′). At the same time, reintroduction of over-expressed Prdx6 without iPLA2 activity was performed by Ser32 gene mutation into the knockdown cells. The iPLA2 assay kit (Xinyu Biological Technology Co, Shanghai, China) and GSH assay kit (Nanjin Jiancheng Bioengineering Institute, Nanjing, China) was used to detect the lentivirus interference efficiency by measuring the iPLA2 activity and GSH activity of Prdx6.

### MTS Assay

Neuron viability was measured by an MTS assay kit (Nanjin Jiancheng Bioengineering Institute, Nanjing, China) to detect any remaining dehydrogenase activity in living cells. After the co-culture system was treated with OGD/R, a total of 10 uL MTS was directly added to the neuron, then incubated for 2 h to allow MTS to metabolize to formazan. The result was analyzed at 490 nm using a microplate reader (Bio-Rad). The neuron viability was expressed as relative percentage compared with control cells. Number of experiments: 9.

### Lactate Dehydrogenase (LDH) Assay

The release of Lactate dehydrogenase (LDH) by microglia was measured using the LDH assay kit according to manufacturer’s instructions (Nanjin Jiancheng Bioengineering Institute, Nanjing, China). Following treatment, the medium of the cell co-culture system from each group was collected and transferred to new 96-well plates, then mixed with the reaction solution provided in the kit. The optical density was measured at 450 nm using a microplate reader (Bio-Rad). Number of experiments: 9.

### Animals and Groups

Adult male Sprague-Dawley rats weighing 270–310 g were bred at and obtained from the Laboratory Animal Center of the Chongqing Medical University. All rats were housed in a colony room with food and water available before the operation under optimal conditions (12/12 h light/dark cycle with humidity 60 ± 5%, 22 ± 3°C). Experimental animals were randomly allocated to the following groups: (1) sham group (Sham); (2) MCAO group (MCAO): underwent MCAO; (3) scramble group (Scramble): treated with scramble of Prdx6 siRNA and subjected to MCAO; (4) Prdx6 siRNA group (Prdx6 siRNA): treated with Prdx6 siRNA and subjected to MCAO; and (5) Prdx6 siRNA + MJ33 group (Prdx6 siRNA + MJ33): treated with MJ33 (0.5 μmol/kg, by tail vein, at 24 h before MCAO) and Prdx6-siRNA and subjected to MCAO.

### Administration of Prdx6 siRNA

Prdx6 siRNA (sense primer 5-CUUCCACGAUUUCCUAGGATT-3 and antisense primer 5-UCCUAGGAAAUCGUGGAAGTT-3) were designed and chemically synthesized by GenePharma Corporation, Shanghai, China. A scramble of Prdx6 siRNA, which has the same nucleotide composition of the target gene siRNA with no sequence homology to any known rat genes, was used as the control. Rats were anesthetized, and 6 μL siRNA was injected into the left lateral cerebral ventricle, siRNA was slowly injected into the left lateral ventricle over a 5 min duration using a Hamilton microsyringe with the coordinates of 1.0 mm posterior to the bregma, 2.0 mm lateral to the midline, and 3.5 mm ventral to the surface of the skull under the guidance of a stereotaxic instrument. After injection, the needle was held in place for 5 min and then removed slowly.

### Middle Cerebral Artery Occlusion Model

The MCAO technique was used to induce transient focal cerebral ischemia according to our previously published methods (Chen et al., 2016; Li et al., 2016). Briefly, ischemia was produced by advancing the tip of a rounded 0.32 mm monofilament nylon suture (Beijing Sunbio Biotech Co. Ltd, Beijing, China) into the left internal carotid artery through the common carotid artery stump and gently advanced to occlude the MCA. After 1 h of occlusion, the thread was withdrawn to restore blood flow. Sham-operated animals received the same procedure, with the left common carotid artery isolated, but not occluded. Rats that did not show neurological deficits after reperfusion (neurological score <2) were excluded from the study, as were animals that died after ischemia induction. Rats that showed neurological deficits immediately after reperfusion (neurological score ≧2) but were found to be experiencing skull-base or subarachnoid hemorrhage were also excluded from the study.

### Evaluation of Neurological Deficit Score

Neurological deficit scores were assessed by an examiner blinded to the experimental groups after 24 h of reperfusion. The deficits were scored on a modified scoring system developed by Longa et al. (1989) as follows: 0, no neurological deficits; 1, failure to extend right forepaw fully; 2, circling to right; 3, falling to right; and 4, does not walk spontaneously and has depressed levels of consciousness. The higher the neurological deficit score, the more severe the impairment of motor motion injury. Nine animals were taken for each group. Brains from these rats were analyzed for water content, infarct volume, ELISA analysis, Western blot, and real-time qPCR.

### Measurement of Brain Edema

Brain water content was measured using the standard wet-dry method. The brains were rapidly removed and dissected into the ipsilateral and contralateral hemispheres. The two hemispheres were immediately weighed on an electronic balance to obtain the wet weight and then heated in an oven at 100°C for 48 h to obtain the dry weight. Brain water content was calculated using the following formula: brain water content (%) = (1 − dry weight/wet weight) × 100%.

### Infarct Volume Analysis

The brain infarct area was evaluated using 2,3,5-triphenyltetrazolium chloride (TTC) staining. TTC was converted to red formazan product in the presence of a functioning mitochondrial electron transport chain. The infarct tissue areas of all coronal sections of each brain were measured using Image-Pro Plus software as described previously. The corrected volume was calculated according to the formula: percentage of infarct volume (%) = [total infarct volume − (left hemisphere volume − right hemisphere volume)]/right hemisphere volume × 100% (Chen et al., 2012).

### ELISA Assay for IL-1β, IL-17 and IL-23

To investigate the level of interleukin cytokine expression, a rat IL-1β, interleukin-17 (IL-17) and interleukin-23 (IL-23) enzyme-linked immunosorbent assay (ELISA) kit (Nanjing Jiancheng; R&D Systems) was used according to the manufacturer’s instructions. Absorbance was determined at 450 nm by spectrometry.

### Real-Time qPCR Analysis

For real-time RT-PCR, the total RNA from primary cultured microglia cells and brain tissues of rats was extracted and purified as previously reported (Yamada et al., 2002). Then the RNA was converted to complementary DNA (cDNA) by reverse transcription (Bio-Rad). Then, cDNA was performed by a PrimeScript^TM^ RT regent kit (TaKaRa Biotechnology, Dalian). The primer sequences (Sangon Biotech, Shanghai, China) were as follows: Prdx6 (forward primer, 5′-ACAgCCCgTgTggTATTCAT-3′, reverse primer, 5′-CTCTCTCCCTTCTTCCAGTCAA-3′), inducible nitric oxide synthase (iNOS; F, 5′-gTgCTAATgCggAAggTCAT-3′, R, 5′-gAAggCgTAgCTgAACAAgg-3′), TLR2 (F, 5′-gAgTCTgCTgTgCCCTTCTC-3′, R, 5′-gCTTTCTTgggCTTCCTCTT-3′), TLR4 (F, 5′-TggCATCATCTTCATTgTCC-3′, R, 5′-CAgAgCATTgTCCTCCCACT-3′), cyclooxygenase-2 (COX-2; F, 5′-GTTGCTGGGGGAAGGAATGT-3, R, 5′-AGAAGCGTTTGCGGTACTCA-3′), nuclear factor-kappa B (NF-κB; F, 5′-ACGACGATCCTTTCGGAACT-3′, R, 5′-TGTTGACAGTGGTATATCTGTTGAA-3′), β-actin (F, 5′-CACCCGCGAGTACAACCTTC-3′, R, 5′-CCCATACCCACCATCACACC-3′). The data were analyzed using a Bio-Rad CFX96 Connect Real-Time system. The mRNA levels were standardized to β-actin, and the values were expressed as the fold change of the threshold cycle value for the control by the 2^−ΔΔCt^ method. Three independent experiments were performed.

### Western Blot Analysis

Proteins were obtained and homogenized in the RIPA buffer. Equivalent amounts of protein (30 μg) were loaded and separated by 10% SDS-PAGE gels, then transferred to polyvinylidene difluoride (PVDF) membranes (0.45 mm). The membranes were washed in Tris-buffered saline containing 0.05% Tween-20 (TBST) followed by blocking for 1 h using 5% nonfat milk in TBST at room temperature, then incubated at 4°C overnight with the following primary antibodies: monoclonaln antibody to Prdx6 (1:1000; ab133348, Abcam, Cambridge, MA, USA), rabbit polyclonal antibody to TLR2 and TLR4 (1:400, BA1716/BA1717, Boster Biological Technology, China), rabbit polyclonal antibody to NF-κB (1:1000; sc-109, Santacruz, CA, USA), rabbit monoclonal antibody to iNOS (1:500, bs0162R, Bioss, China), rabbit monoclonal antibody to COX-2 (1:500, 55070-1-AP, Proteintech, China,) and mouse monoclonal antibody to β-actin (1:10,000, D110007-0100, Songon Biotech, China). The next day, the blots were washed and incubated for 1 h with respective HRP-conjugated secondary antibodies (ZSGB-BIO, Beijing, China; dilution 1:3000) at room temperature. Finally, the protein was visualized using the ECL kit (Millipore, Temecula, CA, USA). The results were quantified using Quantity One 1-D analysis software, and β-actin was used as the internal control. Three independent experiments were taken.

### Statistical Analysis

All data were expressed as mean ± SEM and analyzed using Statistical Package for Social Sciences (SPSS) 16.0 software. One-way analysis of variance (ANOVA) were performed, followed by *post hoc* tests with the least significant difference (LSD; under variance homogeneity). Moreover, Kruskal-Wallis and Welch’s ANOVA were both used for heteoscedastic data. A value of *P* < 0.05 was considered statistically significant.

## Results

### The Efficiency of Prdx6-iPLA2 siRNA

**Figure 1:**
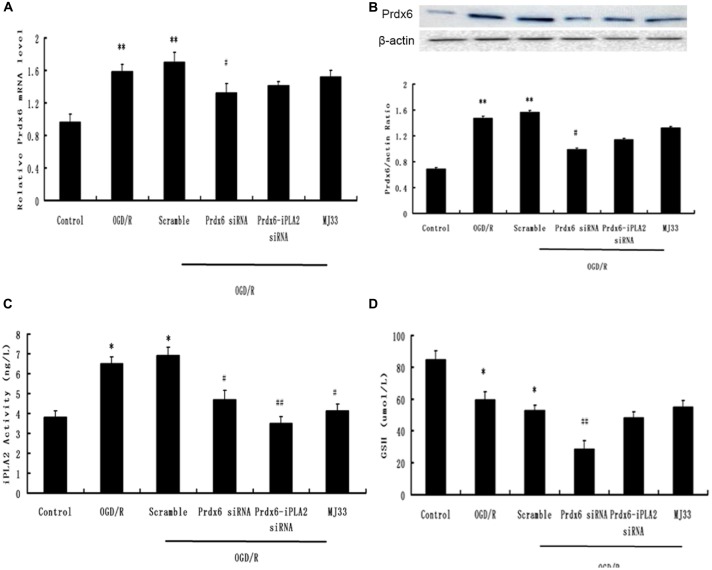
**The efficiency of phospholipase A2 of peroxiredoxin 6 (Prdx6-iPLA2) siRNA.** Effects of siRNA on mRNA **(A)** and protein expression **(B)** of Prdx6. **(C)** An independent phospholipase A2 (iPLA2) enzyme-linked immunosorbent assay (ELISA) kit was used to measure the PLA2 activity in microglia. **(D)** Both Prdx6-iPLA2 siRNA and 1-hexadecyl-3-(trifluoroethgl)-sn-glycerol-2 phosphomethanol (MJ33) had no effect on GSH activity. Values are expressed as mean ± SEM of three independent experiments, *p < 0.05 vs. Control; **p < 0.01 vs. Control; ^#^*p* < 0.05 vs. Scramble, ^##^*p* < 0.01 vs. Scramble.

As mentioned in “Materials and Methods” Section, we used an siRNA and, following functional recovery, performed mediated knockdown of Prdx6-iPLA2 activity. Previous research has shown that Prdx6 siRNA can reduce both iPLA2 and GSH activity (Jo et al., 2013), In order to detect the siRNA efficiency, we added a Prdx6 siRNA group as a control. First, qPCR and Western blotting were used to ascertain whether the Prdx6 mRNA and protein levels were reduced in the microglia (three independent experiments were performed). Figures 1A,B show that considerable reduction in Prdx6 was observed in the Prdx6 siRNA group (*P* < 0.05). No statistical difference in Prdx6 expression was observed between the OGD/R group, Scramble group, Prdx6-iPLA2 siRNA group and MJ33 group. Next, an iPLA2 ELISA kit was used to measure the iPLA2 activity in microglia (Figure 1C). Compared with the Sham group, the iPLA2 activity was increased in the OGD/R group and Scramble group. Treatment with Prdx6-iPLA2 activity siRNA or iPLA2 inhibitors (MJ33) resulted in a significant decrease of iPLA2 activity compared with the Scramble group. Additionally, we detected GSH peroxidase activity (Figure 1D). Prdx 6 siRNA suppressed GSH activity. Both Prdx6-iPLA2 siRNA and MJ33 had no effect on GSH peroxidase activity (*P* > 0.05). All of these results suggest that our methods had interference efficiency of Prdx6-iPLA2 activity in microglial cells.

### Effect of Prdx6-iPLA2 Activity on Neuron Viability and Damage in Response to OGD/R

**Figure 2:**
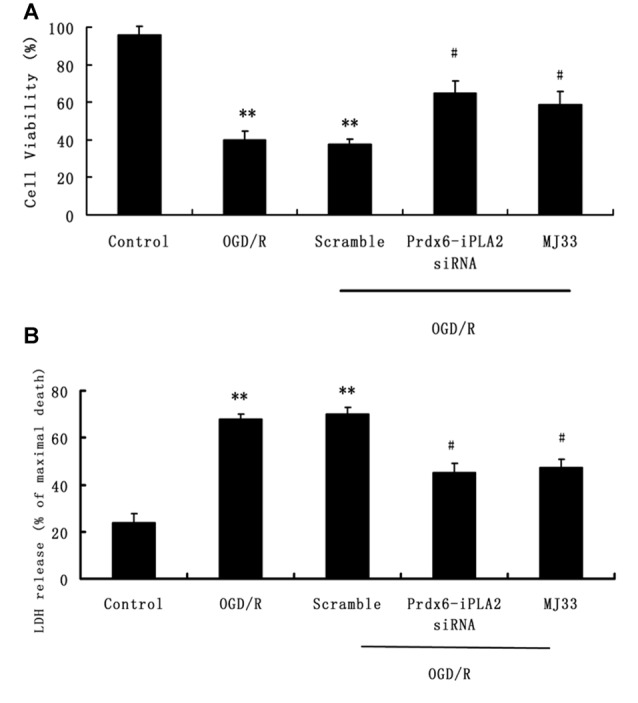
**Effects of Prdx6-iPLA2 on neuron viability and cell damage in response to oxygen glucose deprivation and regeneration (OGD/R). (A)** Neuron viability was measured by MTS assay. **(B)** Neuron damage was measured by Lactate dehydrogenase (LDH) assay. Number of experiments: 9. Values are mean ± SEM, **p < 0.01 vs. Control; ^#^*p* < 0.05 vs. Scramble.

The MTS assay was used to measure the effect of Prdx6-iPLA2 activity on neuron viability after OGD/R exposure (Figure 2A). Cell viability was significantly decreased in the OGD/R group compared with the untreated group (*P* < 0.01). The total number of viable neurons increased to 65 ± 6.4% and 58.8 ± 7% in the Prdx6-iPLA2 siRNA and MJ33 groups, respectively (Figure 2A). In parallel, the release of LDH in neurons was measured (Figure 2B). The siRNA of Prdx6-iPLA2 could decrease the LDH release from neurons compared with the control group (*n* = 9, *P* < 0.05). MJ33 is a fluorinated phospholipid analog that shows relatively tight binding to Prdx6 (Manevich and Fisher, 2005). MJ33 treatment had similar results. These results suggest that the Prdx6-iPLA2 siRNA could reduce neuron damage after OGD/R.

### Effect of Prdx6-iPLA2 Activity on the Release of IL-1β, IL-17 and IL-23 in Culture Medium in Response to OGD/R

**Figure 3:**
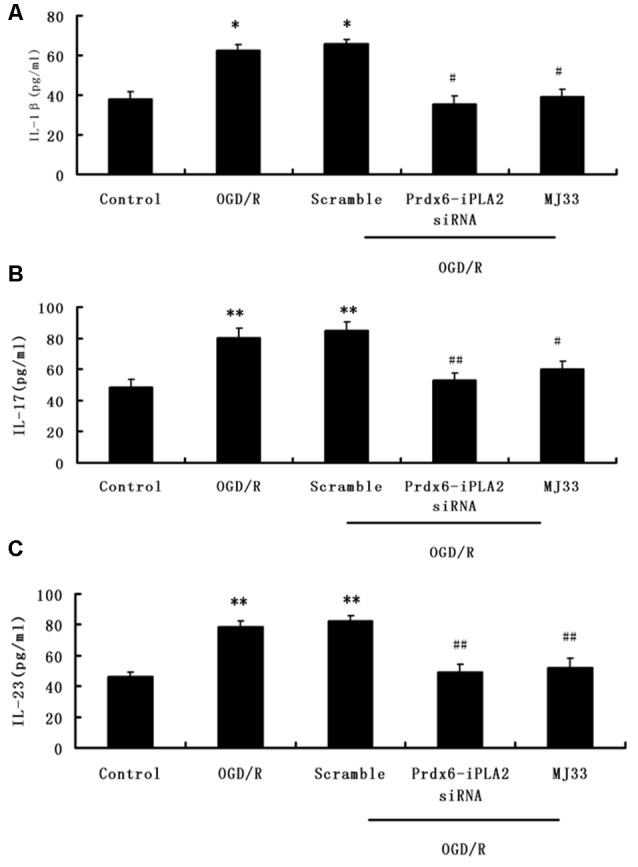
**Effect of Prdx6-iPLA2 on the release of interleukin1β (IL-1β), interleukin-17 (IL-17) and interleukin-23 (IL-23) in culture medium in response to OGD/R (A–C).** Number of experiments: 9. Values are mean ± SEM, *p < 0.05 vs. Control; **p < 0.01 vs. Control; ^#^*p* < 0.05 vs. Scramble; ^##^*p* < 0.01 vs. Scramble.

In order to measure the effects of Prdx6-iPLA2 activity on the expression of inflammatory mediators, ELISA assays was performed. As shown in Figures 3A–C, Prdx6-iPLA2 siRNA significantly decreased the levels of IL-1β, IL-17 and IL-23–35.25 ± 4.2 (pg/ml; Figure 3A, *n* = 9, *P* < 0.05), 53 ± 4.5 (pg/ml; Figure 3B, *P* < 0.01) and 49 ± 5.4 (pg/ml; Figure 3C, *n* = 9, *P* < 0.01), respectively, compared to the Scramble group. MJ33 treatment also decreased these mediators. These results suggest that Prdx6-iPLA2 activity may affect the release of some inflammatory cytokines.

### Effect of Prdx6-iPLA2 Activity on TLR2 and TLR4 Signaling Pathway in Response to OGD/R

**Figure 4:**
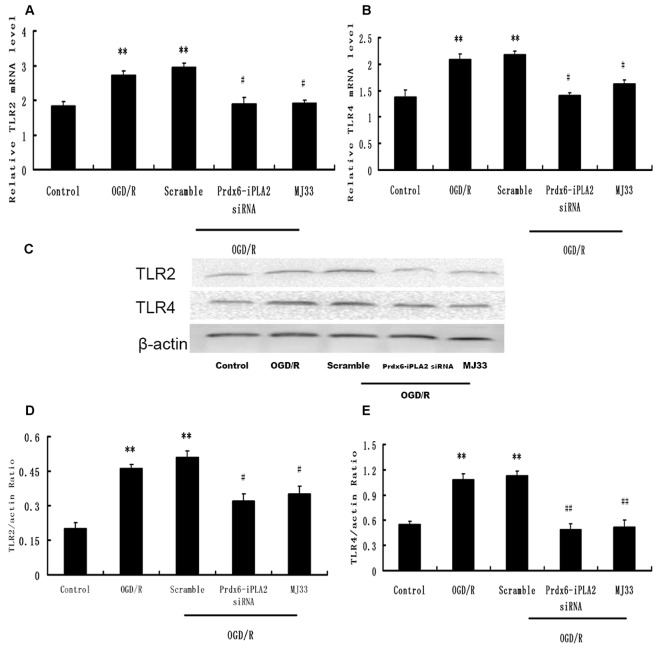
**Effect of Prdx6-iPLA2 on Toll-like receptor 2 (TLR2) and TLR4 expression in microglia in response to OGD/R.** TLR2 and TLR4 were activated during OGD/R exposure. Prdx6-iPLA2 siRNA or MJ33 treatment could inhibit TLR2 and TLR4 mRNA **(A,B)** and protein expression **(C–E)**. Results are expressed as the mean ± SEM of three independent experiments. **p < 0.01 vs. Control; ^#^*p* < 0.05 vs. Scramble; ^##^*p* < 0.01 vs. Scramble.

We next investigated whether the TLR2/4 signaling pathway was involved in the Prdx6-iPLA2-mediated activation of inflammatory mediators. After OGD 6 h/R 24 h, microglial cells were harvested for Western blot analysis. As shown in Figure 4, the Scramble group showed no changes in expression levels of TLR2 or TLR4, while Prdx6-iPLA2 siRNA markedly reduced both the mRNA and protein levels of TLR2 and TLR4, relative to the Scramble group. Similar results were obtained with MJ33 treatment.

### Effect of Prdx6-iPLA2 Activity on the Expression of NF-κB, iNOS and COX- 2 in Response to OGD/R

**Figure 5:**
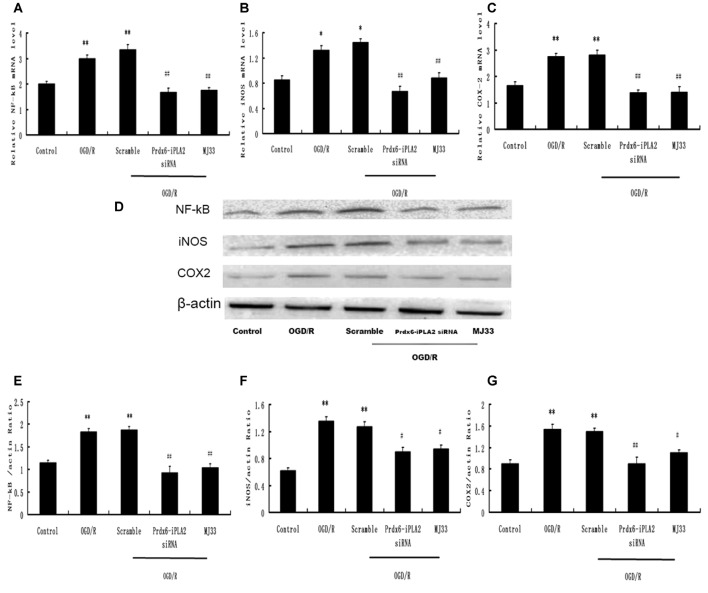
**Effect of Prdx6-iPLA2 on nuclear factor-kappa B (NF-κB), inducible nitric oxide synthase (iNOS) and cyclooxygenase-2 (COX-2) expression in microglia in response to OGD/R. (A–C)** mRNA levels analyzed by quantitative RT-PCR. Representative Western blot bands and semi-quantitative analyses of NF-κB **(D,E)**, iNOS **(D,F)** and COX-2 **(D,G)** expression in astrocytes under OGD/R. Results are expressed as mean ± SEM of three independent experiments. *p < 0.05 vs. Control; **p < 0.01 vs. Control; ^#^*p* < 0.05 vs. Scramble; ^##^*p* < 0.01 vs. Scramble.

The expression of molecules involved in the intracellular TLR2 and TLR4 signaling pathways was detected. The mRNA expression levels of NF-κB, iNOS and COX-2 were decreased by Prdx6-iPLA2 siRNA treatment (Figures 5A–C). In Figures 5D–G, the Western blot results and quantification showed that the protein expression levels of NF-κB, iNOS and COX-2 in the Prdx6-iPLA2 siRNA group decreased by 50.27% (*P* < 0.01), 66.67% (*P* < 0.05) and 58.44% (*P* < 0.01), respectively, relative to the Scramble group. Consistent with previous studies, blocking Prdx6 iPLA2 activity by MJ33 significantly decreased the injury associated with inflammation (Kuang et al., 2014).

### Effect of Prdx6 siRNA and MJ33 on the Expression of Prdx6 and iPLA2 Activity Following MCAO

**Figure 6:**
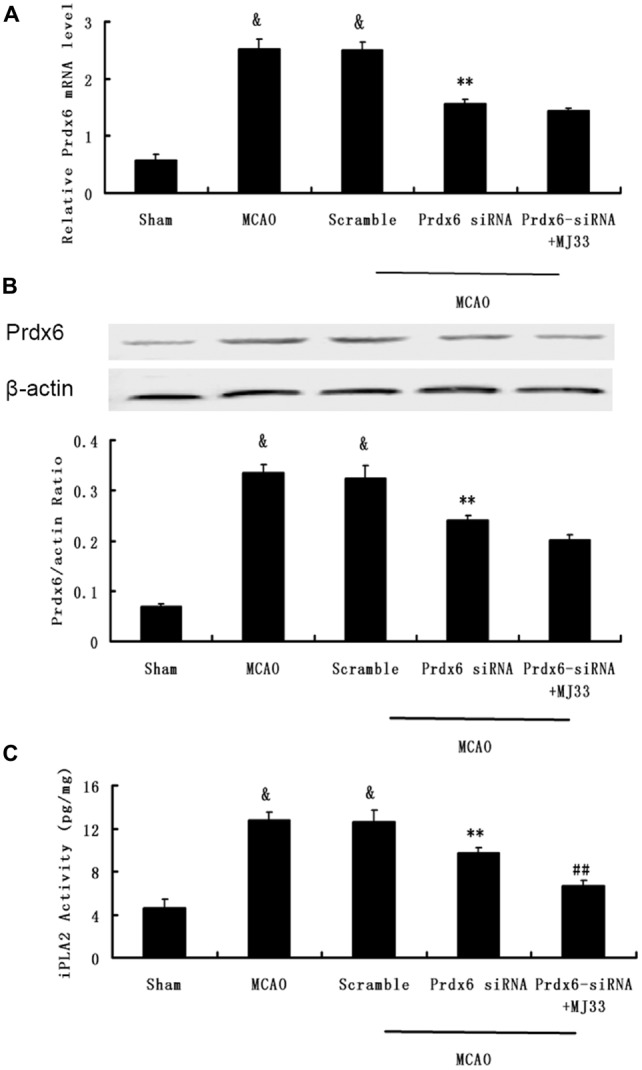
**Effect of Prdx6-siRNA and MJ33 on the expression of Prdx6 and iPLA2 activity following middle cerebral artery occlusion (MCAO).** qPCR **(A)** and Western blot **(B)** results showed that the expression of Prdx6 was reduced in the Prdx6-siRNA group. ELISA **(C)** results also showed that a significant decrease of iPLA2 activity was found in the Prdx6 + MJ33 group. Results are expressed as mean ± SEM of three independent experiments. ^&^*p* < 0.01 vs. Sham; **p < 0.01 vs. MCAO; ^##^*p* < 0.01 vs. Prdx6-siRNA.

We next investigated the role of iPLA2 activity of Prdx6 in transient cerebral ischemia and the potential mechanism underlying the observations. Western blotting and real-time PCR were performed to measure the expression of Prdx6 at mRNA and protein levels (Figures 6A,B). Then, the iPLA2 activity of Prdx6 was detected by ELISA (Figure 6C). Significant decreases in Prdx6 mRNA and proteins levels were observed in the Prdx6 siRNA group. Although additional treatment with MJ33 did not significantly influence the expression of Prdx6 stimulated by Prdx6 siRNA (Figures 6A,B, *P* > 0.05), additional injection with MJ33 resulted in a significant decrease of iPLA2 activity (Figure 6C, *P* < 0.01).

### Effect of Prdx6 siRNA and MJ33 on Cerebral Infarct Volume, Neurologic Deficit Scores and Brain Water Content Following MCAO

**Figure 7:**
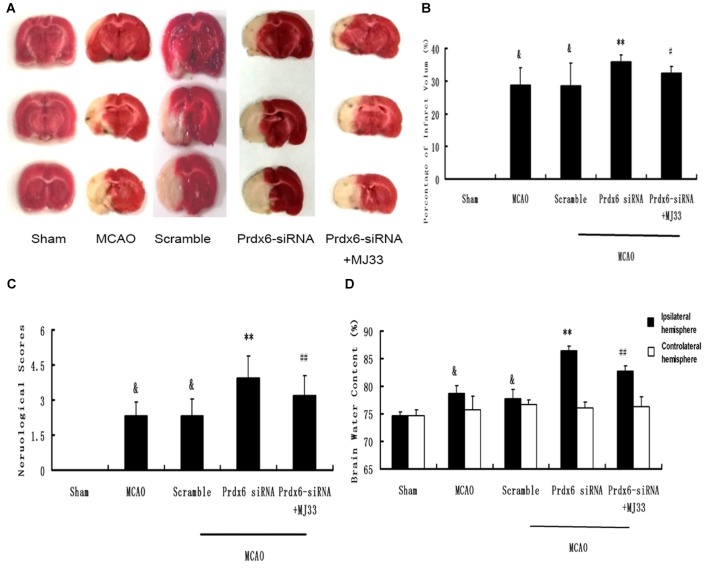
**Effect of Prdx6 siRNA and MJ33 on cerebral infarct volume, neurologic deficit scores and brain water content following transient MCAO. (A)** Representative images of TTC-stained sections 24 h after MCAO. **(B)** Prdx6 siRNA significantly increased cerebral infarct volume compared with the MCAO group. Combine treatment with Prdx6 siRNA and MJ33 attenuated the injury. Neurological function **(C)** and brain water content **(D)** were tested after 24 h of MCAO in rats. Values are mean ± SEM of nine animals in each group. ^&^*p* < 0.01 vs. Sham; **p < 0.01 vs. Scramble; ^#^*p* < 0.05, vs. Prdx6 siRNA; ^##^*p* < 0.01 vs. Prdx6 siRNA.

As shown in Figure 7, compared with the MCAO group, Prdx6 siRNA was associated with significantly aggravated infarct volume (Figures 7A,B, *P* < 0.01). However, this increase was mitigated by additional treatment of the rat with MJ33, an inhibitor of iPLA2 activity. Compared with the Prdx6 siRNA group, similar attenuation was observed in the neurological deficit analysis (Figure 7C, *n* = 9, *P* < 0.01) and brain water content (Figure 7D, *n* = 9, *P* < 0.01) in the Prdx6 siRNA + MJ33 group.

### Effect of Prdx6 siRNA and MJ33 on the Producion of IL-1β, IL-17 and IL-23 in Cortex Following MCAO

**Figure 8:**
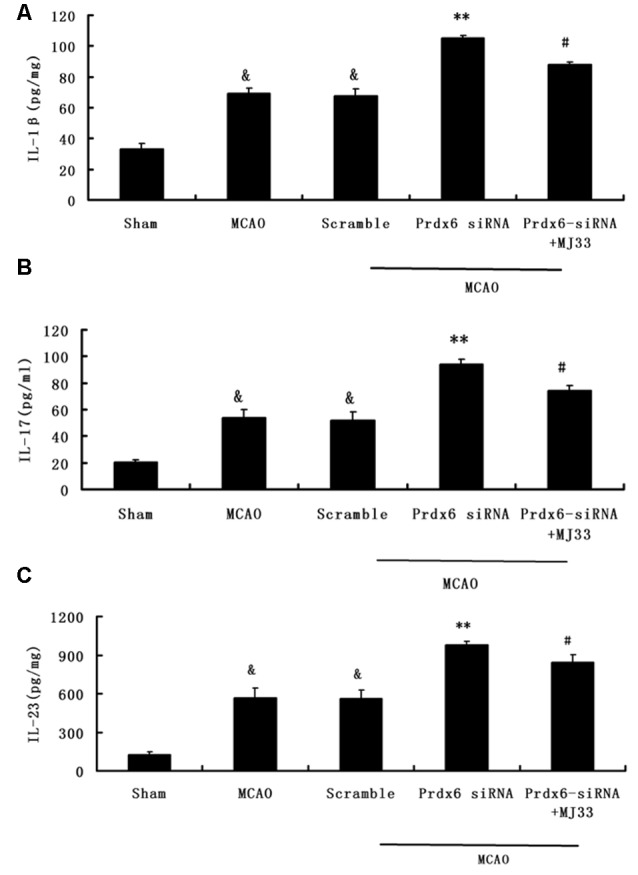
**The influence of Prdx6-siRNA and MJ33 on the production of inflammatory cytokines IL-1β, IL-17 and IL-23.** The ELISA results indicated that IL-1β **(A)**, IL-17 **(B)** and IL-23 **(C)** were upregulated by Prdx6 siRNA after MCAO. These increase were attenuated by additional treatment of rats with MJ33. The results are expressed as the mean ± SEM of nine animals in each group. ^&^*p* < 0.01 vs. Sham; **p < 0.01 vs. Scramble; ^#^*p* < 0.05, vs. Prdx6 siRNA.

To detect the production of IL-1β, IL-17 and IL-23 in brain ischemia/reperfusion, ELISA were performed. As shown in Figure 8, Prdx6 siRNA caused increased release and expression of IL-1β(A), IL-17 (B) and IL-23 (C). However, additional treatment with MJ33 significantly blocked the increase in these inflammatory cytokines in rats stimulated by Prdx6 siRNA treatment.

### Effect of Prdx6 siRNA and MJ33 on TLR2 and TLR4 Expression in the Cortex Following MCAO

**Figure 9:**
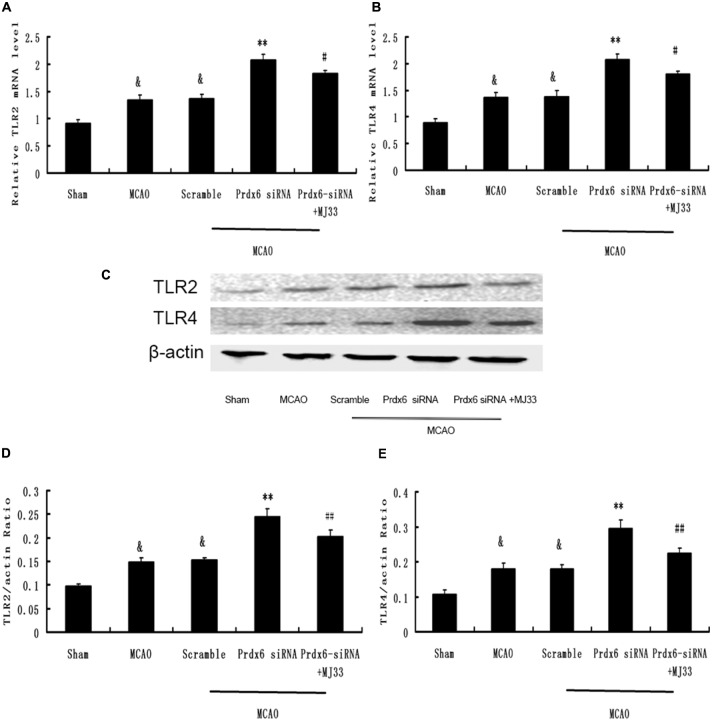
**Expression of TLR2 and TLR4 in response to Prdx6 siRNA and MJ33.** Real-time PCR showed that TLR2 **(A)** and TLR4 **(B)** expression increased after MCAO or giving Prdx6-siRNA. Combined treatment with Prdx6-siRNA and MJ33 downregulated TLR2 and TLR4 levels. Representative Western blot **(C)** and semi-quantitative analyses of the levels of TLR2 **(D)** and TLR4 **(E)** in the cortex after MCAO. Results are expressed as the mean ± SEM of three independent experiments. ^&^*p* < 0.01 vs. Sham; **p < 0.01 vs. Scramble; ^#^*p* < 0.05, vs. Prdx6 siRNA; ^##^*p* < 0.01 vs. Prdx6 siRNA.

To determine the underlying molecular effects of iPLA2 activation *in vivo*, rat cortical tissue from MCA regions was harvested and analyzed by real-time PCR and Western blotting. As shown in Figure 9, the expression of TLR2 and TLR4 in cortex was significantly increased after MCAO. Prdx6 siRNA significantly increased the expression of TLR2 and TLR4 in comparison to the Sham group. Compared with the Prdx6 siRNA group, both the mRNA (Figures 9A,B) and protein levels (Figures 9C–E) of TLR2 and TLR4 were significantly decreased in the same brain areas by combined exposure to Prdx6 siRNA and MJ33.

### Effect of Prdx6 siRNA and MJ33 on the Expression of NF-κB, iNOS and COX- 2 in the Cortex Following MCAO

**Figure 10:**
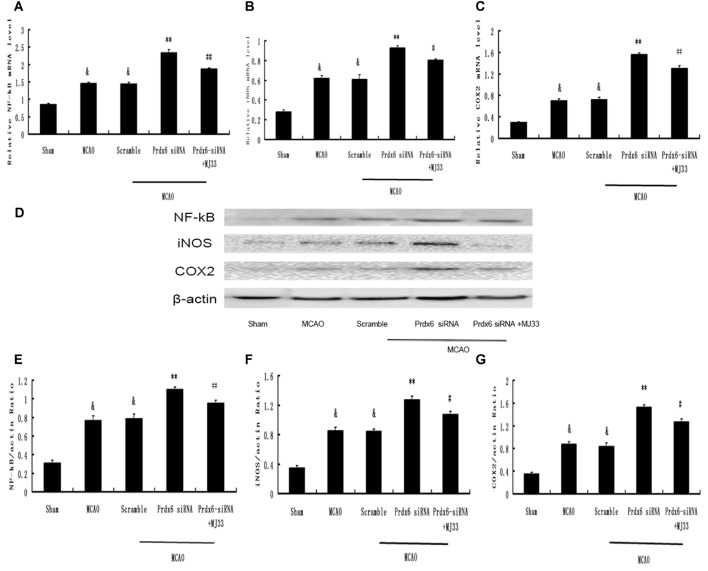
**Expression of NF-κB, iNOS and COX-2 in response to Prdx6 siRNA and MJ33.** Real-time PCR results, representative Western blot bands, and semi-quantitative analyses of NF-κB **(A,D,E)**, iNOS **(B,D,F)** and COX-2 **(C,D,G)** in the cortex following MCAO. Results are expressed as the mean ± SEM of three independent experiments. ^&^*p* < 0.01 vs. Sham; **p < 0.01 vs. Scramble; ^#^*p* < 0.05, vs. Prdx6 siRNA; ^##^*p* < 0.01 vs. Prdx6 siRNA.

We next investigated changes in the mRNA and protein expression of molecules involved in the intracellular TLR2 and TLR4 signaling pathways. MCAO caused increased protein expression of NF-κB, iNOS and COX-2 in the cortex compared with the Sham group (Figures 10A–C). Prdx6 siRNA upregulated the expression of these proteins compared with the control groups (Figures 10D–G). Compared with the Prdx6 siRNA group, combined exposure to Prdx6 siRNA and MJ33 significantly downregulated the mRNA and protein expression of NF-κB, iNOS and COX-2.

## Discussion

In the present study, we explored the function of Prdx6-iPLA2 in cerebral ischemia/reperfusion injury in both OGD/R microglia/neuron co-culture and MCAO models. In the co-culture system, we employed siRNA knockdown and inhibition of Prdx6-iPLA2 activity in the microglia; both resulted in increased neuron survival. We further found that Prdx6-iPLA2 was associated with the secretion of neurotoxic inflammatory mediators (IL-1β, IL-17 and IL-23) and the levels of the TLR2/4 signaling pathway in microglial cells. *in vivo*, consistent with previous reports, the knockdown of Prdx6 by antisense (Prdx6-siRNA) revealed that loss of Prdx6 contributed to increased cerebral infarct volume and poor functional outcomes (Chhunchha et al., 2013; Pan et al., 2014). However, combined treatment with Prdx6 siRNA and Prdx6-iPLA2 inhibitor MJ33 showed a greater diminution in neurologic deficits, cerebral infarction, brain water content and inflammatory molecules than Prdx6-siRNA treatment alone. Thus, the iPLA2 activity of Prdx6 may play a critical role in cerebral ischemia/reperfusion injury, which might target TLR2/4 inflammatory cascades.

Emerging evidence suggests that macrophage/microglia- induced neuroinflammation often precedes and triggers neuronal death in cerebral ischemic disorders. In comparison to monolayer cultures, we established a Transwell co-culture model to better explore microglia-neuron crosstalk. In this system, the two kinds of cells share the same medium and interact through diffusible molecules only. As reported by others, we adopted the Neurobasal medium to meet higher nutrition requirements for neurons (Xing et al., 2013). Before OGD/R treatment, microglia and neurons were co-cultured 24 h for utmost microglial activation. The factors for the harmful inflammatory secreted by microglia have not been clearly defined, but activation of receptors of the innate immune system, including TLR2, TLR4 and their downstream inflammatory cytokines (NF-κB, COX-2 and NOS2) has emerged as a key step in the signaling cascade (Tu et al., 2011; Wang et al., 2013; Lv et al., 2016). Specific molecules blocking TLR2/4 signaling or its endogenous danger-associated molecular patterns (DAMPs) may be a novel therapeutic strategy for post-stroke neuronal inflammation and brain injury (Tu et al., 2010; Kuang et al., 2014; Yu et al., 2015).

Prdx6 is a bifunctional enzyme that harbors iPLA2 (Ca[2+]-independent phospholipase A2) activity in addition to its GSH peroxidase function. Prdx6-iPLA2, rather than other iPLA2 enzymes, was found to be harmful to the cells (Ellison et al., 2012; Krishnaiah et al., 2013). iPLA2 activity of Prdx6 involved in the TNF-induced apoptosis and the proinflammatory response (Kim et al., 2011). In the pulmonary endothelium and alveolar macrophages, Prdx6-iPLA2 activity modulates NOX2 activation via lysophosphatidic acid receptor signaling (Vázquez-Medina et al., 2016). In this study, our focus was the function of Prdx6-iPLA2 activity in the ischemic brain, which is especially at risk for microglia-mediated neuroinflammatory injury associated with TLR2/4 activation.

Single-point deficient mutation by lentivirus gene transduction in primary cerebral cells seemed difficult under our experimental conditions. Here, we described an siRNA and, following functional recovery, performed a mediated knockdown of Prdx6-iPLA2 activity. Finally, we detected successful iPLA2 siRNA with iPLA2 assays. The Prdx6-iPLA2 siRNA showed the same results as MJ33: both had no effect on GSH peroxidase activity (Fisher et al., 1992; Benipal et al., 2015). Previous reports have shown that the iPLA2 activity of Prdx6 is involved in the proinflammatory response (Kim et al., 2011; Shichita et al., 2012a,b). In our study, we found that knockdown of Prdx6-iPLA2 in microglia significantly moderated OGD/R injury to co-culture neurons, which may be associated with decreases in both the RNA and protein levels of TLR2/4 expression. Like other Prdx proteins, Prdx6 proteins have an active region of TLR2/4 activation and are extracellularly released over 12 h after stroke onset, which coincides with the timing of leukocyte infiltration (Shichita et al., 2012a,b). iPLA2 activity of Prdx6 induced the release of arachidonic acid (AA), which is involved in the inflammatory response (Kim et al., 2011). In addition, AA’s upregulation of TLR4 and downregulation of PPARγ may be associated with acute pancreatitis (Mateu et al., 2015). All of these findings suggest that TLR signaling is effected directly or indirectly by Prdx6-iPLA2 activity. Recently, two opposing functions of Prdx6 were shown in brain cells: first, intracellular Prdx6 is thought to be neuroprotective; second, when Prdx6 is released from necrotic cells, it functions as a strong TLR2 and TLR4 stimulator (Shichita et al., 2012a,b; Kuang et al., 2014). It is interesting to note that Prdx6-iPLA2 may be associated with the same inflammation pathway in our present experiment. Because the stimulation of cells may result in Prdx6 binding to the cell membrane, that may activate its iPLA2 activity (Ambruso et al., 2012; Ellison et al., 2012; Krishnaiah et al., 2013). This suggests that there may be a relationship between the Prdx6-iPLA2 and its extracellular danger signal, which requires further study.

In conclusion, our study demonstrated that the iPLA2 of Prdx6 plays important physiological roles in cerebral inflammatory injury both *in vitro* and *in vivo*. siRNA of Prdx6-iPLA2 was accompanied by reduced expression of inflammation cytokines IL-1β, IL-17 and IL-23.The iPLA2 activity of Prdx6 may contribute to neuroinflammation by regulating TLR2/4, leading to the formation of neurotoxic levels of NF-κB, iNOS and COX-2. Our findings provide new insight into Prdx6-iPLA2 function in the brain. Inhibition of Prdx6 iPLA2 activity by gene therapy and/or pharmacological means may constitute a promising new therapeutic approach to the treatment of stroke.

## Author Contributions

YS, JB, TL and ZY conceived and designed the experiments; YS, JB, TL and GM conducted the experiments; YS, JB, TL, LS and PL analyzed the results; GM, LS and PL contributed materials and analysis tools. YS wrote the article; all authors reviewed the manuscript.

## Conflict of Interest Statement

The authors declare that the research was conducted in the absence of any commercial or financial relationships that could be construed as a potential conflict of interest. The reviewer DT and handling Editor declared their shared affiliation, and the handling Editor states that the process nevertheless met the standards of a fair and objective review.
